# A new era in cognitive neuroscience: the tidal wave of artificial intelligence (AI)

**DOI:** 10.1186/s12868-024-00869-w

**Published:** 2024-05-06

**Authors:** Zhiyi Chen, Ali Yadollahpour

**Affiliations:** 1 Experimental Research Center for Medical and Psychological Science (ERC-MPS), School of Psychology, Third Military Medical University, No.30 Gao Tan-Yan Main Street, Shapingba, Chongqing, 400038 People’s Republic of China; 2 Faculty of Psychology, Southwest University, Chongqing, People’s Republic of China; 3 Department of Psychology, University of Sheffield, Sheffield, UK

## Abstract

Translating artificial intelligence techniques into the realm of cognitive neuroscience holds promise for significant breakthroughs in our ability to probe the intrinsic mechanisms of the brain. The recent unprecedented development of robust AI models is changing how and what we understand about the brain. In this Editorial, we invite contributions for a *BMC Neuroscience* Collection on “AI and Cognitive Neuroscience”.

## Main text

Artificial intelligence (AI) describes the simulation of human intelligence machines with the ultimate objective that such machines will achieve the capacity for human-level decision-making and problem-solving [1]. AI-based systems are trained using big data to learn how to perform a task. Then, the systems use the learned knowledge to analyze unknown inputs to produce the desired outcome. During this process, the systems are fed large amounts of training data, and analyze the data to identify patterns, logic, and correlations, then employ these patterns to predict future states. A future state could be progression of disease, diagnosis of a disease, object detection, or traffic detection, for example. Neuroscience deals with the scientific study of the structure and cognitive functions of the brain [2]. Neuroscience and AI are mutually interrelated and benefit one another [2, 3]. Theoretical neuroscience has brought various many distinct improvisations into the field of AI [2, 3]. Biological neural networks have inspired the building of complex deep neural network architectures that have been successfully used in various applications.

Neuroscientists have discovered and deciphered reinforcement learning in humans. The characteristics of reinforcement learning have inspired computer scientists to implement reinforcement learning algorithms in AI-based systems, enabling them to learn complex strategies without explicit instructions. Reinforced learning AI aids the building of complex applications, such as robot-based surgery, self-driving vehicles, medical diagnosis, and gaming applications [3]. In turn, with its ability to intelligently analyze complex data and extract hidden patterns, AI is the perfect choice for analyzing complex neuroscience data. In recent decades, by formulating and emulating neuronal mechanisms, the development of AI has been proliferating faster than ever in and beyond the computational sciences [4, 5]. Despite the fact that the fundamental computations of AI were largely conceptualized from cognitive neuroscience, reciprocally translating AI techniques into neuroscience research is revolutionizing our understanding of the intrinsic brain mechanisms underpinning cognition. By using AI techniques, human continuous languages can be semantically reconstructed and prospectively predicted from non-invasive recorded brain signals (e.g., functional magnetic resonance imaging (fMRI) and magnetoencephalographic (MEG) [6]. Beyond semantic processes, what we are imagining and seeing may be visually decoded and replayed from neural representations by using AI-based CEBRA and stable diffusion models, which are in essence turning “mind-reading” into reality [7, 8]. Recently, the advent of the large-scale language model (LLM) ChatGPT has made a big impact in neuroscience, particularly in AI-based human behavioral simulations, standardized neuroimaging data analysis, and even neurotheoretical validations, fueling further interest in bridging AI and human cognition. In summary, the future of cognitive neuroscience looks promising as we enter a new AI-based era. One of the main benefits of AI in cognitive neuroscience is to develop sophisticated multivariate models for identifying neural co-activation patterns associated with cognitive activities. Rather than probing the statistical dependence between neural responses and cognitive components, AI models explicitly decode what and how neural patterns “direct” cognitive outcomes, thus shedding light on the mechanisms underlying both abstract cognitive entities (e.g., attention, working memory) and complicated cognitive scenarios (e.g., socialization, interpersonal interactions). For instance, AI techniques using multivariate pattern analysis (MVPA) were able to demonstrate how the brain circuits of the prefrontal cortex (PFC) encode working memory processes, as opposed to the dominant hippocampus-hub theory (c.f. [9]). Furthermore, AI models have captured real-world social perception and inter-subject emotional responses by identifying neural patterns involving system-across brain networks, which goes far beyond the traditional model of behavior-brain activity associations (c.f. [10]). Another benefit worthy to be stressed for the supportive role of AI in cognitive neuroscience is that AI models enable individual predictions to be compared with traditional group-averaged analysis. Broadly, the primary strength of AI models is to reason and recognize a generalizable pattern by individual neural features from each participant. Thus, capitalizing on the AI models could achieve case-by-case modeling, technically favoring the practices of precision medicine into healthcare, diagnostics, prognostics and treatments.

On the other hand, as one of the pedestals to guide the mathematical development of computational intelligence, the advances of cognitive neuroscience, in turn, provides impetus to AI evolution. It is widely acknowledged that the rapid developments of modern AI models (particularly in deep learning or deep networks) are largely dependent on our understanding of neural architecture. As indicated by the neural mechanisms of brain information processing, deep learning models, one of the cutting-edge AI technologies, is established by mathematically conceptualizing “the neuron” for a computational element in a hidden layer and calculating “the strength of synapses” for weights between them in AI computations. As a didactic example, the long short-term memory network model (LSTM), a mainstream deep learning method for AI prediction, was precisely developed by new findings in neurocognitive mechanism of memory information processes [11]. Overall, beyond the prominent benefits of AI proliferation for development in cognitive neuroscience as discussed, in-depth understanding of cognitive neuroscience is still fostering the technical advances made in AI optimization/developments.

Nonetheless, despite the enthusiasm for AI systems, significant challenges remain. A notable limitation is the knowledge gap between cognitive neuroscience and AI. While the breakthroughs of AI techniques seem to be inspired by the developments of fundamental advances in cognitive neuroscience, this is not always the case. Therefore, addressing the knowledge gap of translating advances in cognitive neuroscience into AI developments may reap many benefits in the current era of AI. In addition, it is of inevitable contradiction that the primary pursuits between cognitive neuroscience and AI are distinct. Cognitive neuroscience aims to elucidate the neural mechanisms underpinning cognition and demonstrate how neural activities encode information directing behavioral output. Nevertheless, AI researchers aim to improve model performance in predicting outcomes from algorithmic optimizations or technical developments, as opposed to how these adjustments work in biological systems. Indeed, the “blackbox” nature of AI still impedes its practical utility in cognitive neuroscience, a shortcoming yet to be sufficiently addressed [12]. Therefore, “white-box” AI models are needed in future studies. Supporting that, the utilization of XAI (eXplainable Artificial Intelligence) has dramatically increased in and beyond the domain of cognitive neuroscience [13]. Another notable challenge for prioritization is the poor generalizability of certain AI models. Despite the promising contributions of AI-assisted diagnostics in medicine, the current poor generalizability and applicability of the majority of existing medical AI models remains a significant challenge [14]. As such, building generalizable AI models to demonstrate the “intrinsic” neural mechanisms of human cognition is a milestone achievement in the interface of cognitive neuroscience and AI. Lastly, considering the unprecedented developments in the field, scientists and policy-makers have a narrow time window to address the ethical issues raised by such developments. Thus far, neither legal limitations nor scientific consensuses have been reached to protect human beings from the potential harms that AI models may bring about, such as mental privacy violation, machine-generative guideline bias and hostile manipulation, leading to the development of ‘neuroethics’ to guide the challenges we may face ahead.

By quoting answers from ChatGPT, AI tells us that “the synergy between AI and cognitive neuroscience could lead to breakthrough advances in brain research and clinical practice, but has challenges to be overcome, such as overly reliance on correlative data, complexity of neural network, ethic concerns and the lack of standardization” [15]. The aim of this Collection is to create a platform for research and opinions on how cognitive neuroscience and AI could drive and serve as aspirations to each other and to see where we go from here.

## Data Availability

Not applicable.

## Abbreviations

AI: Artificial intelligence
ChatGPT: Chat generative pre-trained transformer
XAI: EXplainable artificial intelligence
fMRI: Functional magnetic resonance imaging
LLM: Large-scale language model
LSTM: Long short-term memory network model
MEG: Magnetoencephalographic
MVPA: Multivariate pattern analysis
